# A Four Element Stringray-Shaped MIMO Antenna System for UWB Applications

**DOI:** 10.3390/mi14101944

**Published:** 2023-10-18

**Authors:** Hüseyin Şerif Savcı

**Affiliations:** 1 Electrical and Electronics Engineering Department, Faculty of Engineering and Natural Sciences, Istanbul Medipol University, Istanbul 34810, Turkey; hsavci@medipol.edu.tr; 2 Informatics and Information Security Research Center (BILGEM), TÜBİTAK, Kocaeli 41470, Turkey

**Keywords:** MIMO antenna system, ECC, gain, efficiency, pattern diversity, ultra-wideband

## Abstract

This paper presents a CoPlanar-Waveguide (CPW)-fed stingray-shaped Ultra-WideBand (UWB) Multiple-Input–Multiple-Output (MIMO) antenna system designed for microwave imaging applications. Featuring a diagonal square with four inner lines and a vertical line at the center from toe to tip with a CPW feed line, the unit antenna element looks like a stingray fish skeleton and is, therefore, named as a stingray-shaped antenna. It offers a bandwidth spanning from 3.8 to 12.7 GHz. Fabricated on a 31mil RO5880 RF teflon substrate with a relative permittivity of 2.2, the proposed antenna has dimensions of 26 × 29 × 0.787 mm${}^{3}$. The maximum realized gain achieved is 3.5 dBi with stable omnidirectional radiation patterns. The antenna element is used in a four-antenna MIMO configuration with an isolation-improving structure at the center. The MIMO system has dimensions of 58 × 58 × 0.787 mm${}^{3}$ with a maximum realized gain of 5.3 dBi. The antenna’s performance in terms of MIMO parameters like Envelope Correlation Coefficient (ECC) and Diversity Gain (DG) is within satisfactory limits for medical imaging applications. Time domain analysis also yields positive results, allowing its integration into a breast phantom tumor detection simulation. The simulation and measurement results demonstrate excellent agreement, making this antenna a promising candidate for microwave imaging and biomedical applications.

## 1. Introduction

Within the realm of wireless communication, ultra-wideband (UWB) antennas have garnered increasing recognition both for millimeter waves and sub-6-GHz systems [1,2]. UWB antennas distinguish themselves from conventional narrowband counterparts by concurrently spanning a vast spectrum of frequencies, transcending the confines of specific frequency bands. This characteristic confers notable advantages in selected wireless communication scenarios. Foremost among these advantages is the superior spectrum efficiency exhibited by UWB antennas [3]. Their ability to transmit data across an extensive frequency range translates to enhanced spectral resource utilization and heightened data throughput for communication or better resolution for sensing. This attribute proves particularly advantageous in environments where radio frequencies are congested and available bandwidth is constrained. UWB antennas’ expansive bandwidth empowers them to attain elevated data rates, making them apt for applications necessitating the swift transmission of substantial data volumes. This communication is standardized as IEEE 802.15.4a/f/z [4]. Some notable features of ultra-widebands are them being a low-power communication, having high-precision ranging, and operation in the license-exempt spectrum. Among several use cases are automobile applications, keyless entry, asset finding, data sharing, secure payment, and high-definition multimedia streaming. UWB antennas discharge low-power, brief-duration pulses, resulting in minimal interference with concurrent wireless devices and systems. This property facilitates the seamless coexistence of UWB technology with established narrowband communication infrastructures, facilitating the seamless integration of UWB devices into extant networks. In addition to their spectral efficiency and low interference propensity, UWB antennas excel in the realm of indoor positioning and localization. Leveraging time-of-flight measurements of UWB pulses, they furnish precise indoor tracking capabilities, proving invaluable in domains like asset tracking, industrial automation, and indoor navigation [5]. Furthermore, UWB signals exhibit remarkable penetration characteristics, traversing obstacles such as walls and furniture while retaining reasonable propagation ranges in challenging environments. This robustness renders UWB antennas well suited for deployment in wireless sensor networks, where reliable communication remains imperative even under adverse conditions. UWB antennas are commonly harnessed in impulse radar applications owing to their generation of short pulses and a broad frequency spectrum. They enable precise radar imaging, ground-penetrating radar, and through-wall imaging, finding essential utility in diverse sensing and imaging domains. Across various domains, UWB antennas find application in wireless USB solutions, wireless sensor networks, radar systems, vehicle-to-vehicle communication, and real-time location systems (RTLS) [6]. Their capacity to operate across an extensive frequency spectrum, coupled with their high data transfer rates, minimal interference susceptibility, and precise indoor positioning capabilities, fits in well with diverse industries and exhibits significant advantages for advanced wireless technology. As technology continues its inexorable evolution, UWB antennas are poised to assume an increasingly pivotal role in shaping the trajectory of wireless communication and beyond [7,8]. In particular, UWB technology holds immense promise in microwave sensing, imaging, and localization applications for medical therapeutic and diagnostic purposes. Its broad bandwidth and emission of short pulses empower precise and high-resolution imaging, rendering it apt for various medical non-invasive and non-contacting test endeavors. In medical imaging, UWB promises detailed, real-time images conducive to early cancer detection and monitoring [9,10]. It affords precise material inspections in non-destructive testing applications, finding relevance in the aerospace and construction industries. UWB’s capacity to penetrate obstacles and deliver accurate imaging capabilities positions it as an invaluable tool for advancing microwave imaging techniques across a gamut of critical applications.

Several UWB antenna systems are developed in the literature [11,12,13,14,15,16]. In [11], a planar monopole antenna is presented, demonstrating an impedance bandwidth spanning from 3.2 GHz to 10.6 GHz. The achievement of an ultra-wideband (UWB) response is primarily attributed to specific design modifications. These adjustments involve changing the radiating patch into an inverted triangular form and adding two meandering I-shaped lines to the front radiating patch. These alterations collectively contribute to the substantial broadening of the antenna’s operational frequency range, enhancing its efficacy for signal transmission and reception over a wide frequency spectrum. To further enhance the UWB response characteristics, a square-shaped slot is incorporated into the ground plane, with its dimensions subject to parametric adjustments. This strategic addition enables the antenna to resonate efficiently across a diverse range of frequencies, enabling its utilization in various applications. It is noteworthy that the proposed antenna’s physical dimensions are relatively compact, measuring 26 mm in width, 29 mm in length, and 0.787 mm in height. This compact form factor renders it well suited for integration into compact and portable electronic devices. However, a noteworthy limitation of this antenna design pertains to its gain characteristics. Specifically, the antenna exhibits significant variations in gain across different frequencies or angles of signal reception. This gain variation has the potential to impact the overall performance and reliability of the antenna, particularly in applications where consistent signal amplification is essential. In [12], an inventive antenna design featuring a compact configuration comprised of tilted square loop frames and incorporating a partial ground plane is introduced. The antenna’s physical realization is achieved on a commercially available FR4 substrate, with precise dimensions measuring 14 mm by 18 mm. Notably, this compact antenna offers a remarkably broad operating bandwidth, spanning from 3.3 GHz to 11.5 GHz. Despite its modest physical size, the antenna demonstrates notable performance characteristics. It achieves a peak gain of 1.4 dBi and provides omnidirectional coverage across its operational bandwidth, which is useful for tasks needing signal reception or transmission from different directions. However, it is important to note that while the antenna is compact and provides good coverage, its gain is relatively modest when considering the entire frequency range it operates in. This means that while it excels in wide frequency coverage and omnidirectional radiation patterns, it may not provide enough amplification for applications requiring higher signal gains. A UWB antenna was presented in [13], taking inspiration from the distinctive Inga Marginata leaf. This antenna was designed by employing the truncated ground plane technique, resulting in a substantial physical form measuring 312 × 121 mm${}^{2}$. Despite the larger physical size, this antenna has a decent performance, boasting an operating bandwidth spanning from 3.8 to 8 GHz, coupled with a gain of 3.63 dB. However, it is worth noting that the antenna’s relatively large dimensions pose challenges when it comes to integrating it seamlessly into RF (radio frequency) circuits. Similarly, in [14], a compact planar antenna is presented for ultra-wideband (UWB) operation. It measures 15 mm × 17 mm × 1.548 mm${}^{3}$ in size with an operation starting from a lowest frequency of 3.8 GHz. This antenna is a planar monopole design and uses a linearly tapered microstrip line for feeding. This feeding method, combined with a partial structure of ground plane, ensures excellent impedance matching for efficient operation across the wide UWB frequency range. The antenna achieves an impressive 9.5 GHz bandwidth, covering frequencies from 3.0 GHz to 12.6 GHz with a return loss of 10 dB. Its fractional bandwidth (FBW) is approximately 122%, indicating its ability to cover a significantly wide frequency spectrum around its center frequency. A key advantage is its compact size, making it suitable for integration into space-constrained electronic devices. However, a drawback is that the antenna predominantly radiates toward the backplane, which may be suboptimal for applications requiring more focused radiation in specific directions. In [15], researchers designed a planar antenna for microwave imaging applications, utilizing negative index metamaterials. The antenna’s compact size was achieved by loading the patch antenna with metamaterial unit cells. These left-handed unit cells, composed of modified split-ring resonators and capacitive-loaded strips, enabled the antenna to exhibit negative permittivity and permeability, crucial for its optimal functioning. In measurements, the antenna performed well from 3.4 GHz to 12.5 GHz, with a 5.16 dBi maximum gain achieved at 10.15 GHz. This design approach was also used in reference [16] to create another metamaterial-loaded small planar antenna, operating within a broader frequency range of 4.23 GHz to 14 GHz.

In UWB MIMO systems, multiple antenna elements are utilized at both the transmitting and receiving sides to enhance communication reliability and capacity. These systems leverage spatial diversity and multipath propagation to effectively counter signal fading and interference, resulting in elevated data rates and enhanced resilience in wireless communication [17,18]. The isolation among MIMO antenna elements is necessary for better performance characteristics. Several techniques and structures have been presented in the literature for isolation enhancement among the radiating elements in MIMO systems [19,20,21,22,23,24,25,26,27]. In [19], authors introduce a compact-sized MIMO antenna system (22 × 26 mm${}^{2}$) for ultra-wideband (UWB) use, focusing on enhancing antenna isolation. They achieve this by integrating two Defected Ground Structures (DGSs) nearby. A T-shaped slot used as a DGS structure extends the antenna’s path and reduces its frequency while suppressing surface currents (4–10.6 GHz). Addressing isolation concerns from 3 to 4 GHz, a line slot structure used as the second DGS structure controls coupling without significantly altering the impedance characteristics of the antenna, thanks to precise ground plane etching. In [20], a two-port UWB MIMO setup with a floral design is presented. This configuration shares a connected ground plane between elements and includes an I-shaped slot in the center. Each antenna element has three ellipse-shaped planar patches that look like the petals of a flower, evenly spaced at 60-degree apart from each other. The added structures serve several purposes: they create multiple resonant frequencies, expand the frequency bandwidth, and enhance isolation between patches by absorbing current and reducing mutual coupling effects. The overall size of the MIMO system is compact at 30 mm × 18 mm, and it maintains an error correction coding (ECC) value of less than 0.08. The excessive coupling between the antenna elements of a MIMO system reduces the efficiency and the diversity gain. Several techniques have been employed for mutual coupling reduction. In [28], artificial neural network models and inverse surrogates modeling are employed to design custom resonators to reduce the mutual coupling in an antenna system. In [21], a four-element UWB system is presented. The isolation among radiating elements is enhanced using a pattern and polarization diversity scheme without using any decoupling structure. The antenna elements are arranged in a plus shape, and the total size of the MIMO system is noted to be 90 mm × 90 mm. The authors of [22] presented the UWB system with enhanced isolation using neutralization lines. Similarly, in [23], a UWB system is presented with frequency selective surface (FSS) as a decoupling structure. The isolation up to 16 dB is increased using this technique. In [24], a four-element MIMO antenna system with two identical slot dipoles and two identical planar monopoles is studied. A couple of inverted L-shaped stubs and an inverted Z-shaped stub are used for isolation enhancement. The antenna operates in the band of 3.1–10.6 GHz with higher than 17 dB isolation between antenna elements. In [25], a four-element MIMO antenna system with half-chopped semi-circular antenna fed by via connected asymmetric feeding scheme is presented. The antenna has an operating band of 2.94–14 GHz. The inter-element isolation of 17 dB over the entire operating band is achieved without using any decoupling structures. In [26], a MIMO antenna system with four triangular monopole elements and neutralization ring (NR) structures is designed. The symmetrical and orthogonal arrangement of monopoles improved the diversity. The system has a 14.2 GHz bandwidth starting from 3.1 GHz. In [27], a four-element MIMO antenna system using two quasi-self-complementary antenna elements with dual polarization and band-filtering characteristics is investigated. An orthogonal arrangement of elements gives an isolation that is better than 20 dB in the operating frequency range of 3–12 GHz.

This research presents a novel CPW-fed stingray-shaped antenna for biomedical applications, especially for tumor detection in soft tissues. The antenna aims for wideband coverage with adequate time-domain characteristics crucial for precise microwave imaging. Experimental results demonstrate effective performance across a broad frequency range from 3.4 GHz to 18 GHz, with a maximum realized gain of 3.6 dBi at 7 GHz, enabling adequate microwave signal boost for capture and transmission. The antenna exhibits low group delay (<1.5 ns) for face-to-face and side-by-side configurations, minimizing pulse distortion critical for microwave imaging. A MIMO UWB antenna system with enhanced isolation (up to 17 dB) is developed. A comprehensive comparison with other UWB antennas in the literature highlights the CPW-fed stingray antenna’s unique advantages and improved performance, demonstrating its potential for practical microwave imaging applications.

**Figure 1:**
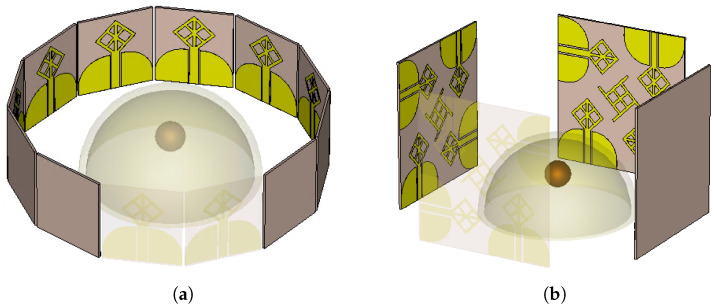
Near-field microwave imaging setup with a tumor inside beast phantom (**a**) with twelve single antenna elements, (**b**) with four MIMO antenna systems.

The performance of the antenna system has a critical role in the overall performance of the near-field microwave imaging systems. In addition to wide bandwidth, the antennas should be able to transmit pulses with little signal distortion. Also, the transient response of the antenna is critical to have proper penetration into the human tissue with minimal signal waveform distortion. This translates into a pulse duration in the order of nanoseconds, which means there is a multi-gigahertz bandwidth requirement for the antenna. The detailed requirements for antenna design specifications are given in [29]. This work proposed a novel structure that improves the isolation and bandwidth without needing to have a connected ground. Such an improvement results in higher resolution detection with less number of antenna elements. Figure 1 depicts a near-field microwave imaging system for breast cancer detection using two different antenna systems. Figure 1a presents twelve stingray antenna elements for 2D detection. The antenna elements are placed next to each other with 30-degree angles, covering the entire phantom under test. Figure 1b presents four MIMO antenna systems with sixteen antenna elements. The spatial diversity of the MIMO system enables 3D detection capability.

The remaining part of this paper focuses on ultra-wideband antenna design for near-field microwave imaging and is organized into five sections. Section 2 starts with the design evolution of the single antenna element, gives simulated and measured s-parameters, and gives time-domain response along with parameters such as system fidelity factor and group delay. Section 3 details the design of the MIMO configuration consisting of four antenna elements, introduces the proposed MIMO configuration with isolating structure and gives the simulation results and surface current densities. Section 4 explains the implementation of the MIMO and its small signal and radiation pattern measurements. The performance parameters such as Envelope Correlation Coefficient (ECC), Diversity Gain (DG), Channel Capacity (CC), and Mean Effective Gain (MEG) are also presented in this section. Section 4 also gives a detailed comparison between the recent literature and the proposed antenna. Finally, in Section 5, the conclusion of this research is summarized by highlighting the outcomes of the study. An additional discussion is added for the projections of the future scope of this work.

## 2. Antenna Design

**Figure 2:**
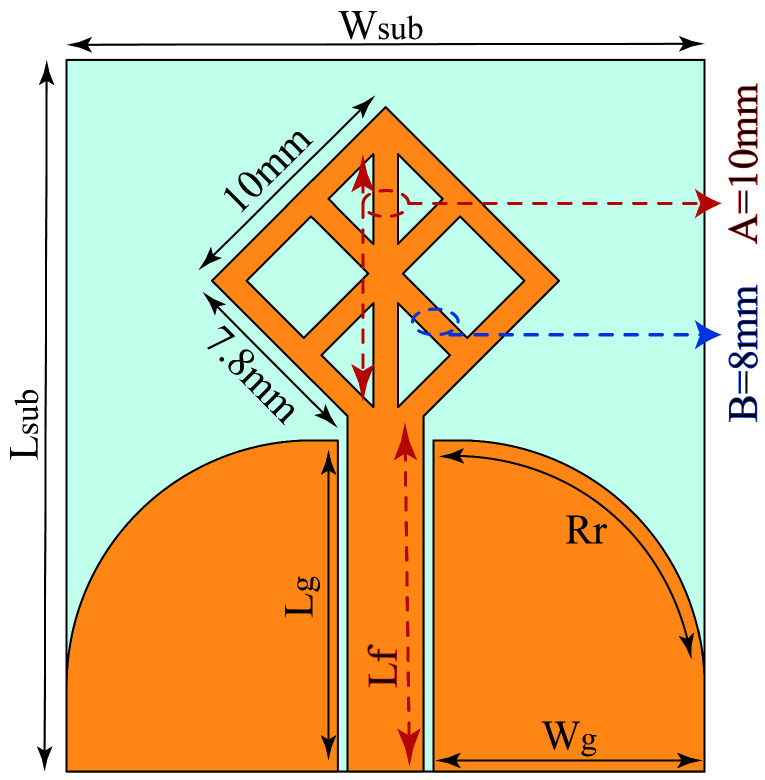
Proposed CPW-fed UWB Antenna.

Figure 2 displays the UWB Antenna design, which utilizes RO5880 material with 0.787 mm thickness. Although there are better options in terms of cost efficiency, the Rogers substrate is chosen due to its low moisture absorption, low dielectric constant, and low dielectric loss properties. Additionally, Rogers substrates can be manufactured with tighter tolerances and thinner layers, allowing for smaller and more precise RF and microwave designs. While Rogers substrates may be more expensive than FR4, their superior performance and reliability make them a preferred choice for microwave imaging experiments. The antenna employs the coplanar waveguide (CPW) feed technique for several reasons, including its low signal losses, inherent coplanar structure, and ease of manufacturing. The proposed antenna dimensions are $W_{Sub}$ = 26 mm, $L_{Sub}$ = 29 mm, $L_{g}$ = 13.5 mm, $R_{r}$ = 10 mm, $L_{f}$ = 14.25 mm, W${}_{g}$ = 11.05 mm, *A* = 10 mm, and *B* = 8 mm. *A* and *B* refer to the width of the vertical line and the diagonal line, respectively.

**Figure 3:**
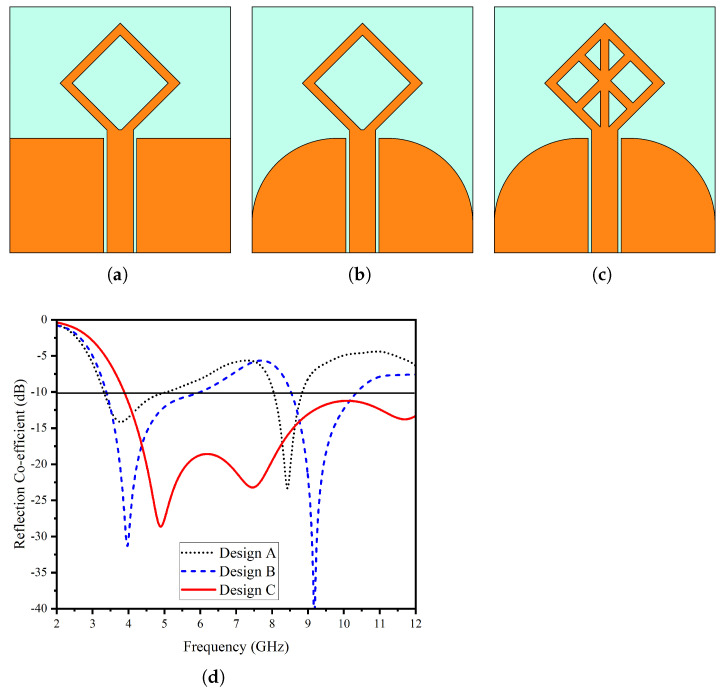
Design evolution (**a**) stage 1, (**b**) stage 2, (**c**) proposed, (**d**) reflection coefficient.

Figure 3 depicts the evolutionary steps. In its initial stage (Stage 1), the configuration featured a feed line with a rectangular coplanar waveguide ground plane and a diagonal square frame as a loop antenna. This setup achieved dual-band resonance, spanning 3–5 GHz and 8–9 GHz, but had unsatisfactory reflection coefficients in the first band, with a more pronounced resonance at 8.5 GHz. In Stage 2, introducing a cFin-shaped ground plane shifted the resonance to lower frequencies, resulting in a wide bandwidth of 3.3–6.5 GHz and 8.5–10.6 GHz. In the final stage (Stage 3), a total of three lines are integrated into the frame (see Figure 3c). Two of them are diagonal lines connecting the top and bottom diagonal outher elements from their midpoints; the third line is a vertical line connecting the top and bottom corners from the tip to toe of the diagonal square. This structure achieves an extended frequency response spanning from 3.8 GHz to beyond 12 GHz.

**Figure 4:**
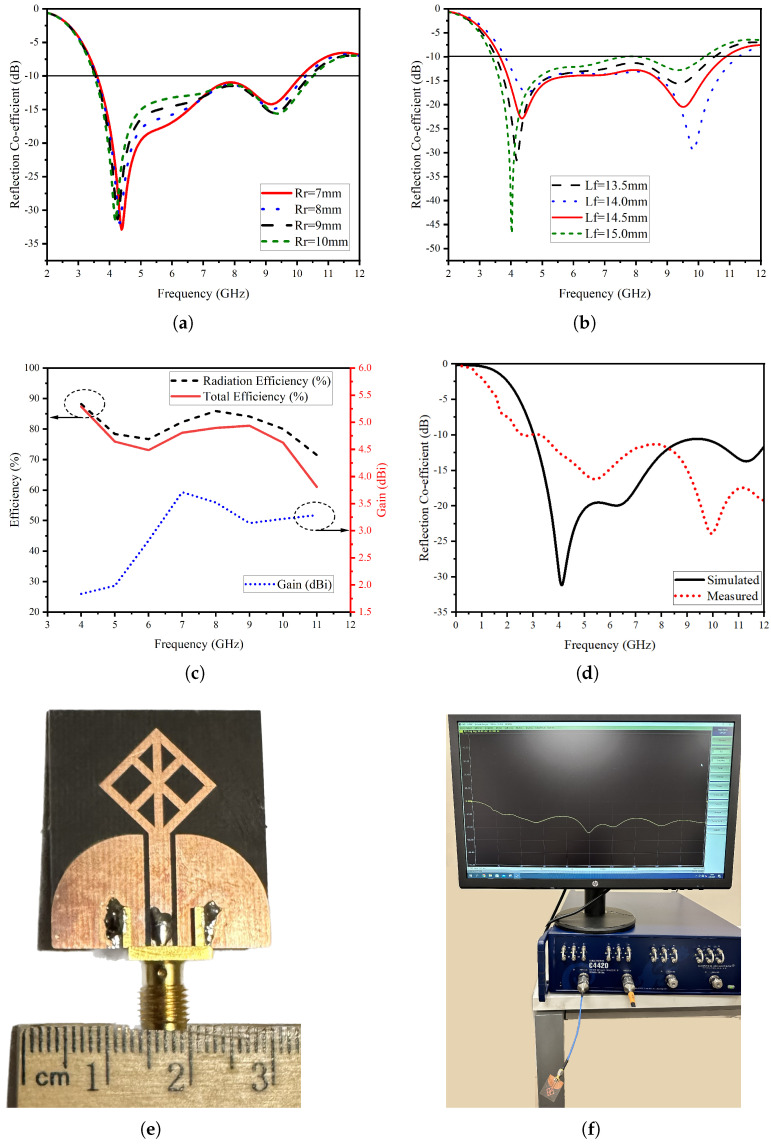
Single antenna element (**a**) parametric analysis of ground arc length, $R_{r}$ (**b**) parametric analysis of feedline length, $L_{f}$ (**c**) radiation efficiency, total efficiency and gain over frequency (**d**) reflection coefficient in dB (**e**) prototype photo (**f**) s-parameter measurement setup.

Figure 4a,b provides the response change to the parametrical analyses pertaining to the dimensions of Rr and Lf. Rr is the length of the arc for the return signal of the CPW structure. The larger value of the length expands the bandwidth by pushing the third resonance towards a higher frequency and pulling the second resonance closer to the first one. This results in two dominant resonances. The Lf is the length of the feedline, which has significant effects on both of the dominant resonances generated by the first step. Upon the parametric analyses, the optimum value for the feedline length is determined as 14.5 mm. Figure 4c provides efficiency along with the maximum gain across various frequencies. Notably, the peak efficiency registers at 7 GHz, reaching a high number of 95%, while the maximum gain reaches 3.71 dBi. The fabricated stingray antenna demonstrates that the measured response of the antenna satisfies the simulated bandwidth requirements, as evident in Figure 4d. The difference in the resonances can be attributed to cables and the antenna’s proximity to the bench during characterization.

### Time Domain Performance

The frequency domain performance of the antenna system has distinctive properties such as gain, polarization, and directivity. However, the specific performance requirements of the ultra-wideband antennas are derived from the time domain analysis, which gives important performance criteria for impulse-driven systems and determines if the system is compliant with the requirements of the intended application. The transient response gives insight into the angles of arrival and departure and polarization.

**Figure 5:**
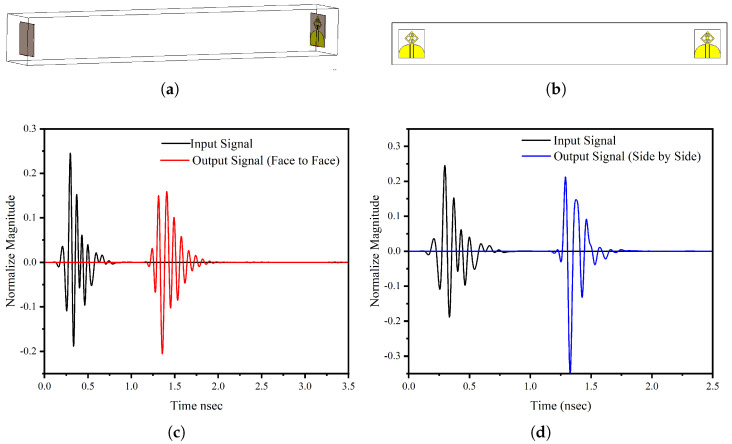
Time-domain response of single antenna element (**a**) face-to-face configuration (**b**) side-by-side configuration (**c**) face-to-face response (**d**) side-by-side response.

The time domain performance of an antenna system is analyzed in the far-field region where the antenna’s radiation pattern is independent of the distance from the antenna. In order to save the computation resource, the antennas should be placed where the far-field region starts. Equation (1) defines the minimum distance where the far-field or the Fraunhofer region starts.

(1) $$R=\frac{2D^{2}}{\lambda }$$

where *R* is the distance from the antenna, *D* is the maximum dimension of the antenna, and λ is the wavelength. The time domain analysis for the face-to-face and side-by-side configurations are analyzed. These two cases are extremes of any multiple antenna configuration for near-field imaging. Depending on the configuration and number of antenna elements, the system may converge, having a side-by-side case. Since the antenna has monopole-type radiation, the side-by-side configuration is also a valid case. A distance of 300 mm is placed between the antennas, which are assigned as transmitting and receiving antennas. The face-to-face and side-by-side configurations are shown in Figure 5a,b. Figure 5c,d demonstrates that both configurations have a peak value of 2 ns showing less distortion.

As the time domain responses show, there is a minimal distortion between face-to-face and side-by-side configurations. This corresponds to a high fidelity factor. The distortion between the radiated and captured signals is given with a cross-correlation value. The highest value of the cross-correlation is defined as the fidelity factor. The fidelity factor is contingent upon the system specifications. It is dependent on the performance parameters of the antenna and the waveform of the excitation signal. The antenna bandwidth should be wide enough to cover the entire pulse spectrum of the excitation signal so that the antenna distortion effect can be properly analyzed [30]. The fidelity factor, which blends an antenna system’s transient and frequency responses, is used to compare the performance of the UWB antennas [31]. The mathematical expression for the fidelity factor is given in Equation (2).

(2) $$F=max\left[\frac{\int_{-\infty }^{+\infty }s_{tx}\left(t\right)s_{rx}\left(t+\tau \right)dt}{\sqrt{\int_{-\infty }^{+\infty }s_{tx}^{2}\left(t\right)dt\int_{-\infty }^{+\infty }s_{rx}^{2}\left(t+\tau \right)dt}}\right]$$

where $s_{tx}$ and $s_{rx}$ are the radiated and captured signals, respectively.

The face-to-face configuration has a higher fidelity factor of −94.5 than the side-by-side configuration of 88.62. A better fidelity factor means lower distortion in the transmitted wave, which is an indicator of a wider bandwidth of the antenna system. The high-fidelity factor antennas are essential for microwave imaging applications. Another important performance parameter of the ultrawideband antennas is the group delay. Group delay is defined as the amount of change in the transmitted signal phase with respect to the frequency change as given in Equation (3)

(3) $$\tau =-\frac{\partial \theta (\omega )}{\partial \omega }$$

where the τ is group delay, θ(ω) is phase of the signal, and ω is the frequency.

**Figure 6:**
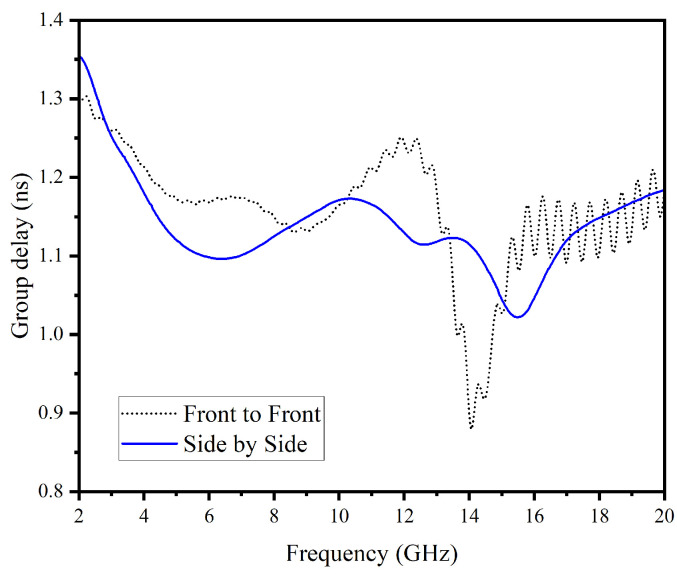
Group delay of UWB antenna.

It varies across the operating spectrum of the antenna system. Microwave imaging systems require a group delay value of less than 2 ns [32]. By assessing the group delay and fidelity factor, the most appropriate configuration of the antenna systems, whether side-by-side or face-to-face, can be determined. From Figure 6, it is evident that the group delay in both configurations exhibits a satisfactory range.

## 3. MIMO Configuration

**Figure 7:**
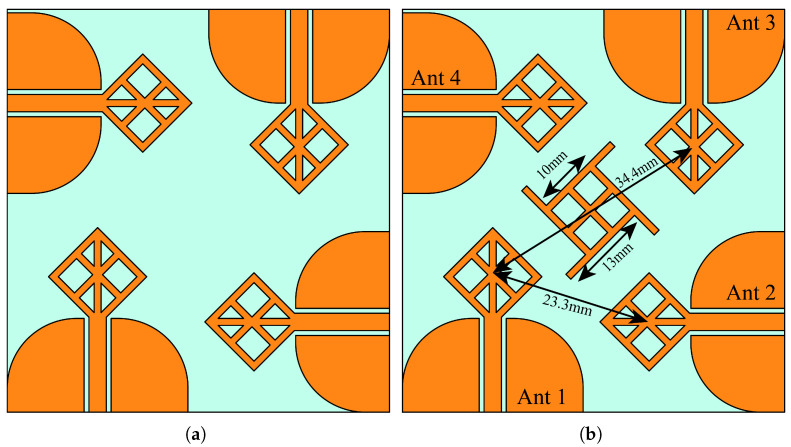
Four element MIMO antennas (**a**) simple configuration (**b**) with isolating structure at the center.

**Figure 8:**
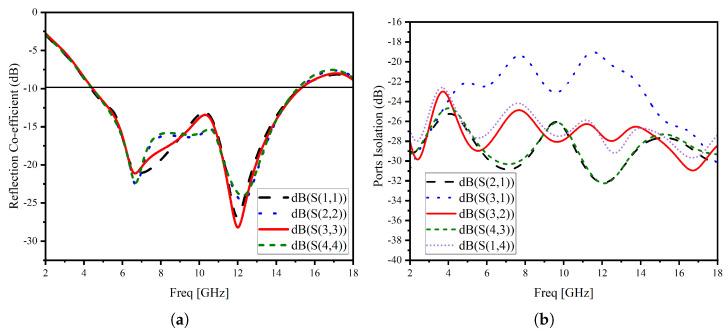

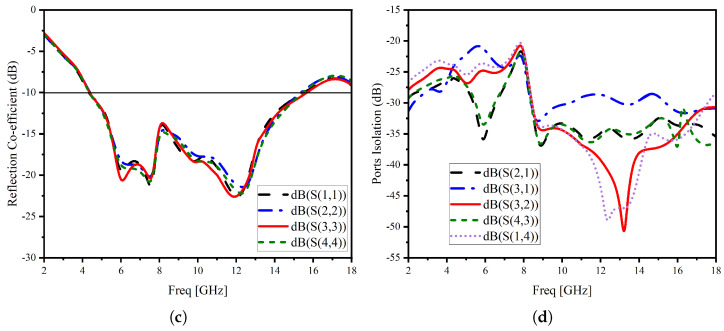
The scattering parameters of four element MIMO antennas (**a**,**b**) simple configuration (**c**,**d**) with isolating structure at the center.

Figure 7 shows two Multiple Input and Multiple Output antennas. Figure 7a is constructed from four stingray-shaped antenna elements with an orthogonal orientation (a 90-degree between antenna elements). This placement and orientation are chosen to allow each antenna to transmit and receive signals with different polarizations and decrease spatial redundancy [33]. Figure 7b is similar to the first MIMO configuration with an additional center structure derived from the single-element antenna, which helps to improve the isolation. The total dimension of the proposed MIMO system is 55 mm × 58 mm${}^{2}$. The four elements of the MIMO antenna have separate grounds. MIMO systems with connected grounds between antenna elements are known to have better isolation characteristics [34]. However, several different structures with and without connected grounds, which have competing performances, are reported in the literature [35,36]. The separate ground MIMO systems often employ various isolation enhancement techniques [37]. Figure 8 illustrates a comprehensive analysis of the S-parameter behavior in our UWB MIMO system, providing invaluable insights into the performance of our antenna array. This figure not only reveals the reflection coefficient response of the resonating elements but also delves into the isolation characteristics. These insights are crucial, shedding light on the system’s behavior both with and without the inclusion of an isolating structure. A pivotal aspect of our UWB MIMO system lies in its ability to maintain a high degree of isolation between its individual radiating elements. Such a high degree of isolation is crucial for enhanced channel bandwidth and minimized radiation pattern degradation for an effective and efficient MIMO system. Here, the isolating element is designed as a window-like structure, which is the same size as the frame of single antenna elements, with four additional stubs at each corner in parallel with the top segment of the corresponding antenna. The spacing between the radiating elements is given as follows. The spacing between the centers of Ant1 and Ant3 is 34.3 mm, whereas between Ant1 and Ant2, it is 23.3 mm. Also, the core size of the isolating structure is the same as antenna elements, except the isolating structure does not have vertical lines. Instead, it has one stub at each of the four corners. The stubs are 3 mm long. The width of all lines in the structure is 1 mm. These stubs tremendously help reduce the unwanted coupling between radiating elements.

The introduction of the isolating structure yields a remarkable enhancement in the isolation response. This enhancement is readily apparent when examining the port isolation values, with notable improvements observed. Depending on the antenna, the isolation values have improved from a minimum of 3 dB up to 20 dB. The isolating structure tremendously improved the isolation, especially between the adjacent antennas, which are antennas 1 and 2, antennas 2 and 3, antennas 3 and 4, and antennas 4 and 1.

**Figure 9:**
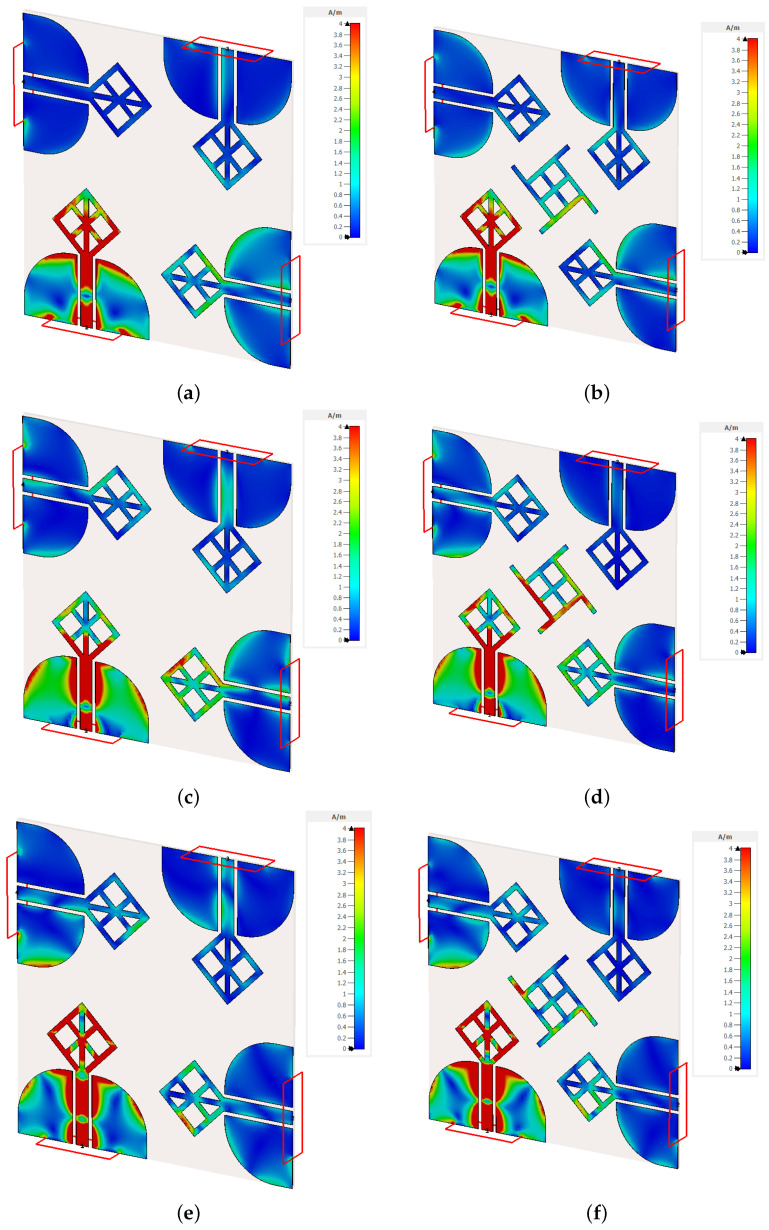
The surface current density for the four elements MIMO antennas (**a**) at 4.5 GHz without isolating element (**b**) at 4.5 GHz with isolating element (**c**) at 6.7 GHz without isolating element (**d**) at 6.7 GHz with isolating element (**e**) at 15.5 GHz without isolating element (**f**) at 15.5 GHz with isolating element.

In Figure 9, an in-depth analysis of surface currents across three distinct frequencies: 4.5 GHz, 7.4 GHz, and 9 GHz. Blue region represents the current densities close to 0, red represents regions with current densities 4 A/m or more. Our investigation focused on a single active antenna element while maintaining impedance-matched terminations for the remaining elements.

It was an interesting observation when non-radiating elements showed noticeable electric currents on their surface in a simple MIMO structure. This was due to the coupling of electromagnetic fields from the radiating elements to the nearby non-radiating ones. This would manifest itself as a reduction in bandwidth and cause distortion. Nonetheless, after a series of experiments, a practical solution in the form of a decoupling structure is employed to reduce these interactions. Many types of simpler truncated polygon structures have been evaluated as decoupling elements. Finally, a unique isolating structure that was derived from the antenna itself is evaluated. The shape and even the length of its corner stubs had a significant effect on the isolation performance. None of the previous experiments were as good as this unique structure for isolating the MIMO antennas. Upon its integration, a noteworthy transformation became evident. Specifically, at 4.5 GHz (Figure 9b), the coupled currents elegantly converged towards the outer regions of the decoupling structure, indicative of resonant behavior at a lower frequency, substantiated by the accompanying data.

Similarly, at 7.4 GHz (Figure 9d), the currents were effectively suppressed, owing to the concerted contributions of both the middle and side sections of the decoupling structure, operating synergistically to restore equilibrium within the coupled electromagnetic fields. Transitioning to the higher frequency of 9 GHz (Figure 9f), the decoupling structure demonstrated its full efficacy, with the middle section taking prominence in proficiently mitigating the induced currents. These empirical observations seamlessly corroborated the supporting findings in s-parameter analysis, thereby underscoring the pivotal role played by the isolator.

## 4. Results and Discussions

**Figure 10:**
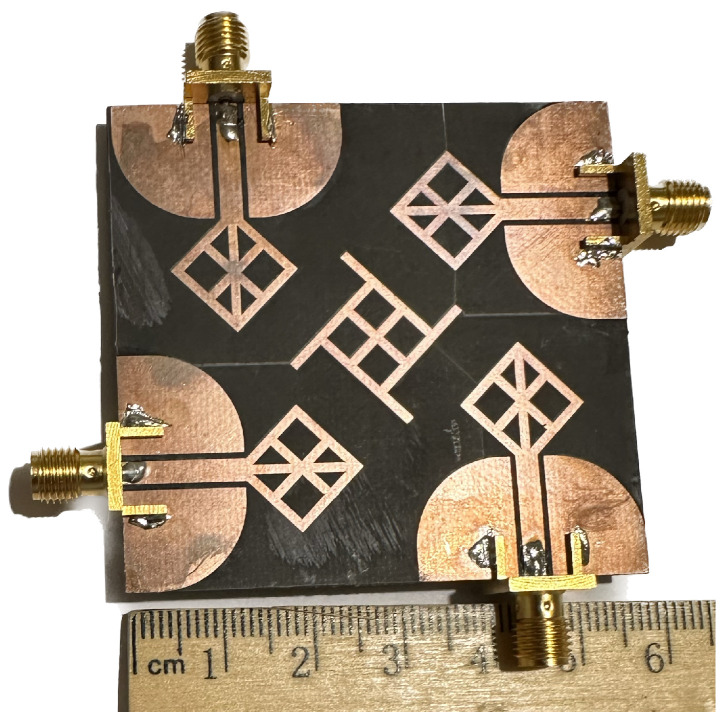
Four-element MIMO antenna system with isolating structure at the center.

**Figure 11:**
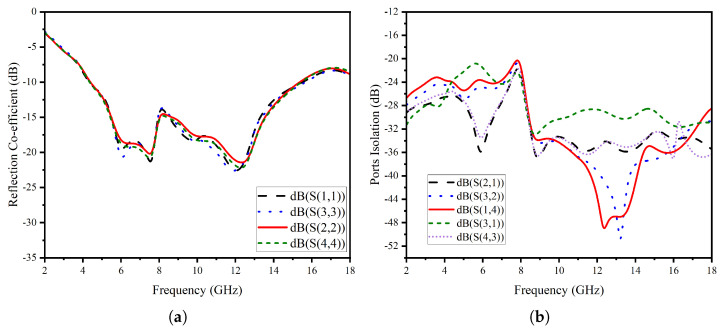
(**a**) Measured reflection coefficient, (**b**) Measured port-to-port isolation.

The proposed stingray MIMO antenna system is fabricated and tested using the in-house facility. Figure 10 shows the fabricated prototype. The antenna’s thin and flexible substrate poses a challenge in keeping it steady during measurements. However, in Figure 11a,b, you can see the measured results of the reflection coefficients and coupling characteristics.

The small differences between the isolation performance of the reciprocal ports of the MIMO antenna system can be attributed to calibration drift, measurement uncertainty, and measurement performance shifts caused by the small bends due to the antenna’s flexibility. Nonetheless, despite the difficulty of keeping these under control, the measured isolation performance is quite successful, reaching a minimum of 22 dB, as shown in Figure 11b. While the measured isolation value is slightly lower before 8 GHz, it still demonstrates excellent performance.

**Figure 12:**
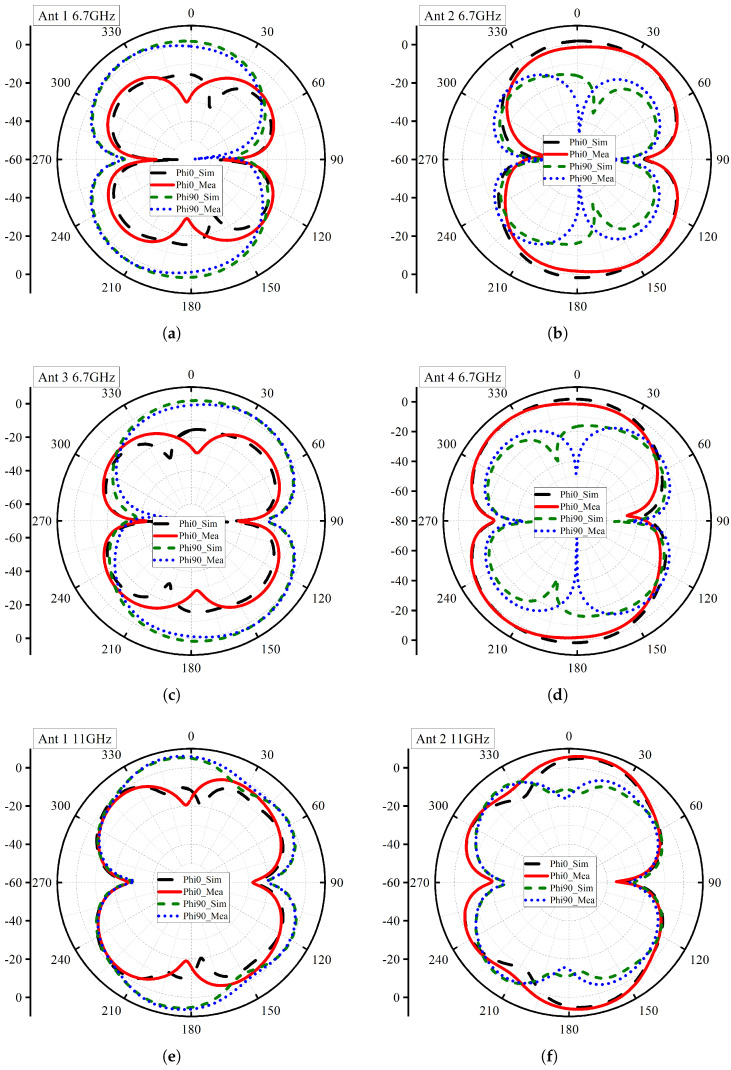

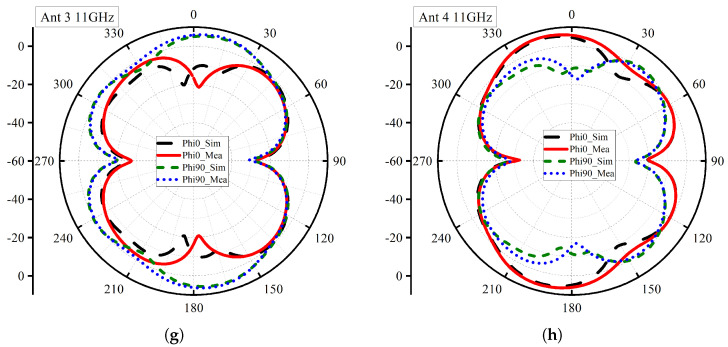
2D Radiation Patterns (**a**) 6.7 GHz Ant 1, (**b**) 6.7 GHz Ant 2, (**c**) 6.7 GHz Ant 3, (**d**) 6.7 GHz Ant 4, (**e**) 11 GHz Ant 1, (**f**) 11 GHz Ant 2, (**g**) 11 GHz Ant 3, (**h**) 11 GHz Ant 4.

The simulated and measured 2D radiation pattern of the proposed stingray MIMO antenna at two different frequencies of 6.7 GHz and 11 GHz are shown in Figure 12. The analyses of ports individually give a comprehensive insight into the antenna’s behavior across the band. The ultra-wideband antennas generally target radiation patterns with non-directional behavior. At 6.7 GHz (Figure 12a), the antenna exhibits monopole-like behavior in the *xz*-plane, by having some gain suppression at two locations. Meanwhile, in the *yz*-plane, the pattern has some level of gain suppression from all four directions. It is evident that radiation in the vertical direction is more efficient.

At higher frequencies, such as 11 GHz (Figure 12e), the antenna gives more performance with an omnidirectional-like pattern in the *xz*-plane. This pattern ensures broad coverage in the horizontal plane, making it suitable for various communication and sensing applications. It is compulsory to highlight that the radiation pattern results present a good agreement between simulation and measurement data despite the mechanical challenges faced during the measurements. This agreement validates the accuracy and practicality of the design, proves the success of implementation, and confirms its performance over the distinct frequencies across the band.

**Figure 13:**
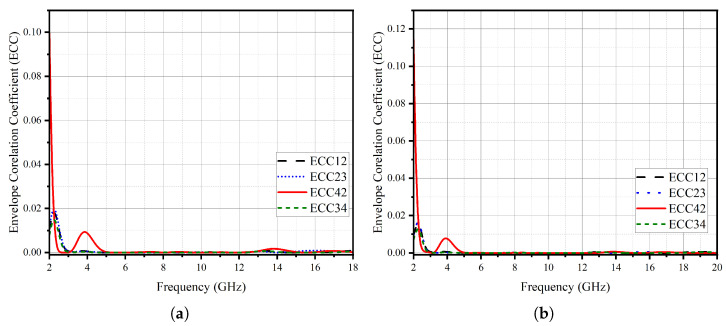

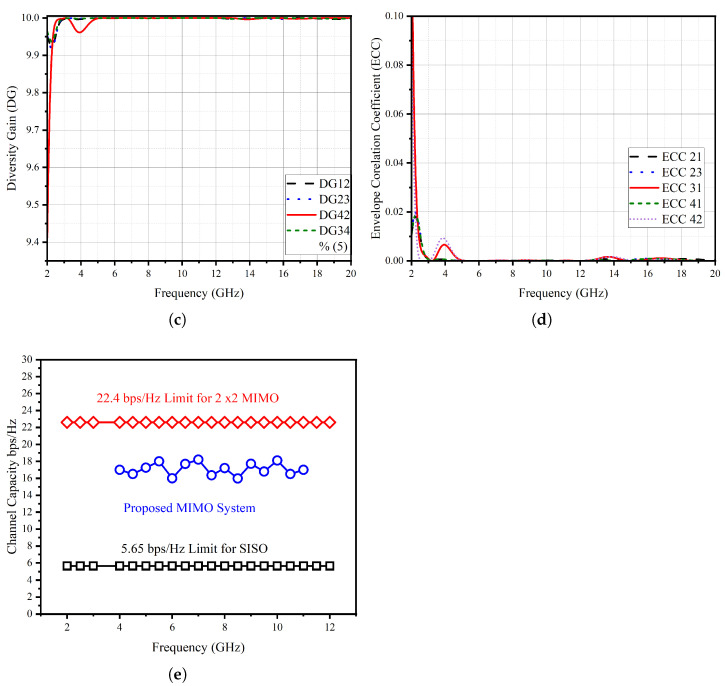
MIMO parameters: (**a**) ECC simulation (with ISO), (**b**) ECC measurement (with ISO), (**c**) DG (with ISO), (**d**) ECC (without ISO), (**e**) CC (with ISO).

The MIMO performance parameters are shown in Figure 13. The ECC is noted to be less than 0.12, and DG for all cases is nearly equal to 10, hence satisfying MIMO performance limits. Furthermore, the peak channel capacity (CC) in Figure 13e is noted to be 19 bps/Hz. The MEG of the proposed stingray-shaped MIMO system is calculated using the following equation below and mentioned in Table 1.

(4) $$MEG=\int_{-\pi }^{\pi }\int_{0}^{\pi }\left[\frac{r}{r+1}G_{\theta }\left(\theta ,\phi \right)P_{\theta }\left(\theta ,\phi \right)+\frac{1}{1+r}G_{\phi }\left(\theta ,\phi \right)P_{\phi }\left(\theta ,\phi \right)\right]sin\theta d\theta d\phi$$

where $G_{\phi }\left(\theta ,\phi \right)$ and $P_{\theta }\left(\theta ,\phi \right)$ are angle of arrival and *r* is the cross-polarization ratio. It can be expressed as Equation (5).

(5) $$r=10log_{10}\left(\frac{P_{vpa}}{P_{hpa}}\right)$$

The $P_{vpa}$ and $P_{hpa}$ represent the received power by the vertically polarized and horizontally polarized antennas, respectively.

Table 2 and Table 3 show the performance comparisons of the published literature and the proposed stingray UWB antenna as a single element and as a MIMO system, respectively. The size comparison is given as physical and electrical sizes, where the dimensions represent the m-th and n-th multiples of the wavelength, $\lambda_{0}$, of the first resonant frequency. The fractional bandwidth (FBW) is also inserted in both tables for better comparison. Table 2 shows that the proposed antenna would be quite good for the compactness of its radiating element. However, the coplanar waveguide-based feed network enlarges the occupied area. It is seen from Table 3 that the proposed MIMO system has excellent isolation performance among the peers, thanks to the novel isolating element at the center of the MIMO structure. If a figure of merit is defined with the multiplication of the number of elements and operating bandwidth; the absolute value of isolation in dB is in the numerator; and the multiplication of total area and the envelope correlation coefficient is in the denominator, it is evident that our proposed antenna would have a high figure of merit.

## 5. Conclusions

This study presents a CPW-fed stingray-shaped UWB antenna element and its orthogonal-oriented four-port MIMO antenna implementation without a novel isolating structure. The single antenna element has a 26 mm × 29 mm while the MIMO structures have 58 mm × 58 mm size. By incorporating an isolating structure, the isolation performance is improved up to 16 dB. The simulation and experimental results exhibit a good agreement, affirming the accuracy of the simulations and the precision of the practical implementation. The measurement results show that the MIMO antenna had a bandwidth of 10 GHz covering a frequency range of between 4.4 GHz and 14.4 GHz. The overall gain of the antenna system is 5.3 dBi; the typical isolation between the antenna elements is 22 dB. Performance metrics pertinent to Multiple-Input–Multiple-Output (MIMO) systems, such as the Envelope Correlation Coefficient (ECC), Mean Effective Gain (MEG), Channel Capacity, and Diversity Gain (DG), are compliant with the requirements of the target applications. The ECC is less than 0.01, while the MEG is calculated between −3 and −4 for all antenna elements across the frequency. Furthermore, a thorough evaluation of the antenna systems in the time domain reveals that they are well below the acceptable thresholds. The proposed MIMO antenna design holds considerable promise for integration into MIMO-based medical imaging systems, where antenna efficiency plays a pivotal role. Elevated levels of isolation, with the proposed novel isolating structures, promise to enhance the precision of medical imaging significantly. The design also offers a powerful solution that can enhance the robustness of wireless connections. This enables smooth data transfer and meets the growing needs of modern consumer electronics. Exploiting the benefits inherent in UWB MIMO technology, this antenna system is positioned to substantially enhance the user experience, marking a new era in unlicensed wireless applications over a range of ultra-wideband frequencies. These applications cover various areas, including personal area network wireless connections, radar systems, microwave imaging, and beyond. The future scope of this work will be extended to a system with more MIMO elements integrated with a feeding network realized with cost-effective materials.

## Figures and Tables

**Table 1:** MEG of the proposed antenna system.

Frequency (GHz)	MEG1	MEG2	MEG3	MEG4
4	−3.78	−4.02	−3.88	−3.76
6	−3.45	−3.81	−4.00	−3.69
7	−2.99	−3.03	−3.13	−3.37
9	−2.87	−3.85	−3.77	−3.25

**Table 2:** Comparison of stingray-shaped single antenna element with the literature.

Ref	Band (GHz)	FBW (%)	E-Size m$\lambda_{0}$ × n$\lambda_{0}$	Size (mm${}^{2}$)	Gain (dB)	Efficiency (%)
[11]	3.6–11.5	105	0.16 × 0.17	13 × 14	1.4	56
[12]	3.2–10.6	107	0.25 × 0.28	23 × 26	4	86
[13]	3.8–8	71	3.95 × 1.53	312 × 121	3.9	76
[14]	3.8–12	104	0.19 × 0.23	15 × 18	2.5	90
[15]	3.4–12.5	109	0.18 × 0.24	16 × 21	5	85
[16]	4.23–14	107	0.15 × 0.22	10.5 × 15	5	85
Prob.	3.8–12.7	108	0.33 × 0.36	26 × 29	3.6	90

**Table 3:** Comparison of proposed MIMO Antenna system with the literature.

Ref	MIMO Elements	Band (GHz)	E-Size m$\lambda_{0}$ × n$\lambda_{0}$	Size (mm${}^{2}$)	Isolation (dB)	ECC
[21]	4	2.77–12	0.83 × 0.83	90 × 90	>19	<0.01
[23]	4	3.1–10.6	0.46 × 0.46	45 × 45	>16	<0.01
[24]	4	2.94–14	0.39 × 0.39	40 × 40	>16	<0.03
[25]	4	3.1–12	0.72 × 0.42	70 × 41	>16	<0.013
[26]	4	3.1–17.3	0.77 × 0.77	75 × 75	>14	<0.0013
[27]	4	3.1–12	0.52 × 0.52	50 × 50	>14	<0.003
Prob	4	4.4–14.4	0.81 × 0.81	58 × 58	22	<0.01

## Data Availability

All the data is available in the study.
